# DeepHLApan: A Deep Learning Approach for Neoantigen Prediction Considering Both HLA-Peptide Binding and Immunogenicity

**DOI:** 10.3389/fimmu.2019.02559

**Published:** 2019-11-01

**Authors:** Jingcheng Wu, Wenzhe Wang, Jiucheng Zhang, Binbin Zhou, Wenyi Zhao, Zhixi Su, Xun Gu, Jian Wu, Zhan Zhou, Shuqing Chen

**Affiliations:** ^1^Institute of Drug Metabolism and Pharmaceutical Analysis and Zhejiang Provincial Key Laboratory of Anti-Cancer Drug Research, College of Pharmaceutical Sciences, Zhejiang University, Hangzhou, China; ^2^College of Computer Science and Technology, Zhejiang University, Hangzhou, China; ^3^MOE Key Laboratory of Contemporary Anthropology, School of Life Sciences, Fudan University, Shanghai, China; ^4^Department of Genetics, Development and Cell Biology, Iowa State University, Ames, IA, United States

**Keywords:** deep learning, neoantigen, recurrent neural network, human leukocyte antigen, cancer immunology

## Abstract

Neoantigens play important roles in cancer immunotherapy. Current methods used for neoantigen prediction focus on the binding between human leukocyte antigens (HLAs) and peptides, which is insufficient for high-confidence neoantigen prediction. In this study, we apply deep learning techniques to predict neoantigens considering both the possibility of HLA-peptide binding (binding model) and the potential immunogenicity (immunogenicity model) of the peptide-HLA complex (pHLA). The binding model achieves comparable performance with other well-acknowledged tools on the latest Immune Epitope Database (IEDB) benchmark datasets and an independent mass spectrometry (MS) dataset. The immunogenicity model could significantly improve the prediction precision of neoantigens. The further application of our method to the mutations with pre-existing T-cell responses indicating its feasibility in clinical application. DeepHLApan is freely available at https://github.com/jiujiezz/deephlapan and http://biopharm.zju.edu.cn/deephlapan.

## Introduction

Cancer cells are different, through somatic mutations, from normal cells and can therefore be recognized being a foreign cell by the immune system. Among all foreign elements in cancer cells, neoantigens are the most widely recognized elements derived from mutated genes (1). Neoantigens have therefore been acknowledged as ideal targets for cancer immunotherapies, such as cancer vaccines and T-cell immunotherapies (2–5). Recent studies also indicated that neoantigens are closely related to the therapeutic effect of immune checkpoint blockade therapies (6–8). However, only a small number of somatic mutations can generate neoantigens. It is still challenging to identify somatic mutations that can generate effective neoantigens (9).

Recently, whole exome sequencing combined with bioinformatic prediction has been widely used for candidate neoantigen identification (10). Several computational pipelines, such as TSNAD (11), pVAC-Seq (12), and INTEGRATE-neo (13), have been developed for this purpose. The most critical component of these pipelines is the *in-silico* estimation of binding between human leukocyte antigens (HLAs) and peptides. Previous prediction methods on peptides binding with HLA alleles can be categorized into three groups, including (i) position-specific scoring matrix (PSSM)-based methods (14, 15), (ii) machine learning-based methods (16, 17), and (iii) structure-based methods (18, 19). There are also some consensus methods that combine several methods for better predictive performance (20, 21). Several neoantigen databases have also been developed based on pan-cancer immunogenomic analyses using neoantigen prediction tools, such as TSNAdb (22) and TCIA (23). However, existing tools are insufficient for neoantigen prediction in clinical applications because few of the predicted binders are immunogenic (1). Many attempts have been made to improve the prediction accuracy. For instance, several researchers have performed epitope prediction based on mass spectrometry (MS) profiling of HLA-peptide sequences. This method also considered proteasomal cleavage and transporter-associated with antigen processing (TAP)- mediated peptide transport, which are necessary in antigen presentation (24–27). In addition, the developers of NetMHCpan have released their latest version, which is trained based on both binding affinities and MS data of HLA-peptide binding (17). Moreover, the rapid development of deep learning methods, such as Convolutional Neural Networks (CNNs), has led to an increase in their use in cancer immunology (28–32). However, current methods will never catch up to the need for clinical applications unless they consider the potential immunogenicity of presented mutant peptides complexed with HLA class I molecules (pHLA). Several studies have considered the binding of pHLA and T-cell receptors (TCRs) to select immunogenic neoantigens using a neoantigen immune fitness model, which assumes that the TCR recognition probability of pHLA is positively correlated with the similarity between mutant peptides and pathogenic antigens (33–35). Furthermore, Jurtz et al. trained a sequence-based predictor of the interaction between TCRs and peptides presented by HLA-A02:01, which considers the sequences of both TCRs and peptides (36).

In this study, we develop a novel Recurrent Neural Network (RNN)-based approach, named DeepHLApan, for neoantigen prediction, considering both the binding between HLA-peptide pairs and the potential immunogenicity of pHLA. DeepHLApan consists of two models: the binding model for predicting the probability of the peptide being presented to the tumor cell membrane by HLA and the immunogenicity model for predicting the potential of pHLA eliciting T-cell activation. Since there are much more binding data than immunogenicity data, we take the prediction score of the immunogenicity model as a filter and rank the prediction scores of the binding model for high-confidence neoantigen identification. The binding model of DeepHLApan achieves comparable performance with other well-known binding prediction tools on the latest Immune Epitope Database (IEDB) benchmark and an independent MS dataset. We also confirm that the immunogenicity model could significantly improve the performance of other neoantigen prediction tools from previously published work (improvement ranging from 20.5 to 55.4%). We further applied our method to mutations with pre-existing T-cell responses and ranked most of them (69%) in the top 20, under an expression threshold of transcripts per million (TPM) >2. These results indicate that DeepHLApan, combining the binding model and the immunogenicity model, could be beneficial for high-confidence neoantigen prediction and could further contribute to tumor immunotherapy in practice.

## Materials and Methods

### HLA-Peptide Binding and Immunogenicity Data

The binding data between HLA class I alleles and peptides were collected from the IEDB (http://www.iedb.org/) (37). HLA-peptide pairs were filtered using the following criteria: (1) HLA class I alleles that are of HLA-A, B, and C subtypes, (2) the length of peptides range from 8 to 15, and (3) the pairs with inconsistent experimental results are excluded. In total, we obtained 327,178 non-redundant HLA-peptide pairs covering 169 HLA alleles with 257,089 of them being binders (Figure S1A, Table S1). We then balanced the positive data and negative data of each allele by following these steps: (1) train a basic model based on the 327,178 pairs, (2) create pseudo-HLA-peptide pairs. The number of created pseudo pairs of each HLA allele *N*_*pse*_ was calculated as follows:

(1) $$\begin{array}{r} N_{pse}=100*|N_{pos}-N_{neg}| \end{array}$$

where *N*_*pos*_ and *N*_*neg*_ represent the number of positive and negative pairs of each HLA allele, respectively. Each peptide is generated by selecting one protein in the Ensembl database (38) randomly and extracting 8–11 mer peptides randomly in this protein without mutations, and (3) predict the binding possibility of pseudo pairs using the basic model and selecting the high-confidence negative (score <0.1) or positive (score >0.9) pairs for each allele.

After these steps, most of the alleles had balanced data, and the alleles with unusual proportions ($\frac{N_{pos}}{N_{neg}}>5\;or\;\frac{N_{neg}}{N_{pos}}>5$) were removed (Figure S2, Table S2). Finally, 437,077 HLA-pairs covering 81 HLA alleles were used for training the binding model, with 280,525 collected pairs and 156,552 pseudo pairs (Figure S1B, Table S3).

The immunogenicity data of pHLA were also retrieved from the IEDB (37). The criterion for data filtering is as follows: (1) The length of peptides range from 8 to 15, and (2) for pairs with inconsistent experimental results, we selected the positive pairs. Finally, we obtained 32,785 HLA-peptide pairs with 5,702 of them related to HLA-A02:01 (Table S4). Among the 32,785 HLA-peptide pairs, 7,212 of them are immunogenic and 3,013 immunogenic HLA-peptide pairs are related to HLA-A02:01.

### IEDB Benchmark Data

The data used to test the performance of the binding model were derived from the IEDB weekly benchmarking website (http://tools.iedb.org/auto_bench/mhci) (version 2018-05-11). It should be noted that this benchmark dataset includes 14 sub-datasets, but three of them with SLA molecules from *Sus scrofa* were not suitable for the binding model and were thus excluded. We then renumbered the selected 11 sub-datasets for convenience, and the datasets measured by binding affinity were transformed to the binary type under the threshold as IEDB stated: HLA-A03:01 is 602 nM, HLA-A02:01 is 255 nM, HLA-B07:02 is 687 nM, and HLA-B27:05 is 584 nM. To note, all the data in this benchmark dataset were not included in previous training data. Meanwhile, the prediction performance of 12 models on these datasets was downloaded from the IEDB for comparison.

### Independent MS Dataset

We collected an independent dataset from Mei et al. (39) for further binding model evaluation. The dataset has never been used for training in all previous developed tools as the authors claimed, but some of them have been used in the training of the binding model of DeepHLApan. We removed the sub-datasets that have been used for training, 15 sub-datasets (all of them are MS data) were retained and used for the model comparison.

### Neoantigen Dataset Used for Evaluating Immunogenicity Model

The data used for evaluating the immunogenicity model were collected from Koşaloǧlu-Yalçın et al. (40), containing 64 neoantigens with 6,400 random peptides that were generated based on mutation data extracted from The Cancer Genome Atlas (TCGA) database. In their work, NetMHCpan achieved excellent performance based on the AUC under rank thresholds of 10 and 2%.

### CD8^+^ T-Cell Epitopes

We collected 2,023 assayed single-nucleotide variants from 17 patients, including 26 mutations with pre-existing T-cell responses from Bulik-Sullivan et al. (27), which combined the data from four published works (41–44) and substituted RNA-Seq data from tumor-type-matched patients of TCGA.

For the mutations from Tran et al. (41), Gros et al. (42), and Zacharakis et al. (44), all 8–11 mer peptides covering the mutations were extracted for prediction and resulted in 59,726 peptides and 372,252 HLA-peptide pairs (Table S5A). Normally, there would be 8 8-mer, 9 9-mer, 10 10-mer, and 11 11-mer peptides for each mutation; however, some of the mutations are located at the start or end of proteins and possess fewer than 38 peptides. For the mutations from Strønen et al. (43), the provided 2,852 HLA-peptide pairs were used for prediction (Table S5B). To evaluate the possibility of a mutation eliciting T-cell activation predicted by DeepHLApan, we removed the HLA-peptide pairs with predicted immunogenic scores <0.5. We then summed the binding scores of the rest of the pairs with the following formula for a mutation rank within one patient.

(2) $$\begin{array}{r} \mathrm{Pr}\left(mutation\right)=\underset{i=8,9,10,11}{\sum }\frac{\sum BS_{imer,h}*r_{imer,h}}{n_{imer}} \end{array}$$

where *Pr(mutation)* is the probability of the mutation presentation, *BS*_*imer, h*_ is the predicted binding scores of the i-mer peptides with HLA h, *r*_*imer, h*_ is the actual ratio of i-mer peptides binding with HLA h in the training dataset, and *n*_*imer*_ is the number of i-mer within one mutation.

The mutation rank data of EDGE and MHCflurry were derived from Bulik-Sullivan et al. (27). The mutation rank of NetMHCpan 4.0 was measured by taking the minimum predicted rank across all mutation-spanning peptides.

We also collected 31 validated immunogenic HLA-peptide pairs from Tran et al. (41), Gros et al. (42), and Stronen et al. (43), which were not provided by Bulik-Sullivan et al. (27) (Table S6). Here, we separated the minimal epitopes according to their length and ranked them. Pairs with predicted immunogenic scores <0.5 were ignored, and the remaining pairs were ranked by the binding score.

### Recurrent Neural Networks

The model architecture used for training was stacked by three layers of bidirectional Gated Recurrent Unit (BiGRU) with an attention layer. A Gated Recurrent Unit (GRU) is a variant of RNN which was first proposed by Cho et al. (45). Similar to the RNN, GRU handles the variable-length sequence by having a recurrent hidden state whose activation at each time is dependent on that of the previous time. The difference between GRU and RNN is the update of the recurrent hidden state which is the core part for overcoming gradients vanish or explode in training model to capture long-term dependencies. In particular, GRU proposes to derive the vector representations of hidden states *h*_*t*_ for each time step t as follows:

(3) $$\begin{array}{r} z_{t}=\sigma \;\left(W_{z}x_{t}+U_{z}h_{t-1}+b_{z}\right)\\ r_{t}=\sigma \;\left(W_{r}x_{t}+U_{r}h_{t-1}+b_{r}\right)\\ h_{t}=\left(1-z_{t}\right){○h}_{t-1}+z_{t}○\phi \left(W_{h}x_{t}+U_{h}\left(r_{t}○h_{t-1}\right)+b_{h}\right) \end{array}$$

where *x*_*t*_ is the input vector, *h*_*t*_ is the output vector, *z*_*t*_ is the update gate vector, *r*_*t*_ is the reset gate vector, *W, U* and *b* are parameter matrices and vector, σ is the logistic sigmoid function, and ϕ is a hyperbolic tangent.

As for the BiGRU, it splits the neurons of a GRU into two directions, one for positive time direction (forward states), and another for negative time direction (backward states). By using two time directions, input information from the past and future of the current time frame can be used.

### Attention Module

The attention module was incorporated with the BiGRU module to make modeling of long-term dependencies of 49 amino acids easier. The attention was first proposed by Bahdanau et al. (46) but we used the type of attention proposed by Raffel and Ellis (47). Given a model which produces a hidden state *h*_*t*_ at each time step, attention-based models compute a “context” vector *c* as the weighted mean of the state sequence h by

(4) $$\begin{array}{r} {e_{t}=\sigma_{a}\left(h_{t}\right),\alpha }_{t}=\frac{exp\left(e_{t}\right)}{\overset{T}{\underset{k=1}{\sum }}exp\left(e_{k}\right)},c=\sum_{t=1}^{T}\alpha_{t}h_{t}. \end{array}$$

where σ_*a*_ is a learnable function which only depends on *h*_*t*_.

### Model Training

Before training, the HLA alleles were transformed into pseudo-sequences as presented by the NetMHCpan (48) method for pan-allele prediction (i.e., each HLA allele was transformed into 34 amino acid residues located within 4.0 Å of the peptide). Then, the peptides were concatenated with the HLA pseudo-sequences. If the length of the combined sequence was <49 amino acids, pseudo amino acid “X” would be used for padding. The one-hot method was used for amino acid representation (i.e., transform each amino acid into a unique vector of 20 zeros and one 1).

Two steps were applied for reliable model training. First, the original dataset was used for basic model training. Then, the preliminary model was used for selecting high-confidence pseudo positive/negative HLA-peptide pairs. The selected pseudo pairs were added to the original dataset to balance the training data for training the final model. Other parameters are as follows: dropout rate was set to 0.2, the sigmoid function was used as the activation function, Binary Cross Entropy (BCE) was employed for loss calculation, and an Adam optimizer with a default learning rate of 0.001 was used for parameter optimization.

### 5-Fold Cross-Validation

Five-fold cross-validation is used to evaluate model robustness. Before training, the dataset is randomly partitioned into five non-overlapping subsets. The cross-validation process is repeated five times, with each subset used as a validation set while the remaining subsets are used as the training set. The results of the five validation sets are averaged to obtain the final result. One hundred epochs are executed, and the model is saved if the validation accuracy is better than previous epochs.

### Evaluation Indicators

The area under the receiver operating characteristic curve (AUC) is the main measurement for model and software comparison. Accuracy (ACC) is used for the performance evaluation of the binding model on single-labeled HLA alleles. Precision [true positives/(true positives + false positives)] and recall [true positives/(true positives + false negatives)] are used to illustrate the importance of the immunogenicity model.

### Data and Software Availability

All datasets for this study are included in the manuscript and the **Supplementary Files**. The source codes of DeepHLApan are freely available at https://github.com/jiujiezz/deephlapan. The web service of DeepHLApan is available at http://biopharm.zju.edu.cn/deephlapan.

## Results

### The Architecture of DeepHLApan

**Figure 1:**
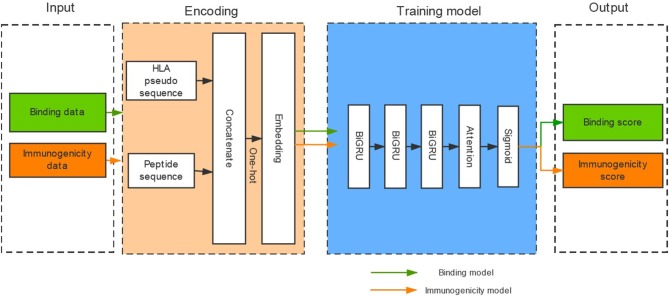
The architecture of DeepHLApan. Two types of data (437,077 binding data points, 32,785 immunogenicity data points) are collected for model training, and one-hot encoding is used for amino acid representation. Three layers of bidirectional GRU with attention have been employed as the model framework. The immunogenic score is used as a filter (>0.5), and peptides with binding scores ranked within the top 20 are predicted as high-confidence neoantigens.

Binding affinity is the most widely used measurement for HLA-peptide binding. However, a large number of HLA-peptide binding data without binding affinity have emerged with the development of high-throughput methods (e.g., MS) for HLA-peptide binding detection. In the binding dataset collected from the IEDB, 66.8% of the binding data were not measured by binding affinity (Table S1). And the gap further increases over time. Therefore, we transformed the binding data with binding affinity into a binary model to create a model that only provides the result of binding or not-binding. In addition, the potential immunogenicity of presented pHLA is a necessary factor for tumor immunotherapy. However, published tools rarely consider the immunogenicity of pHLA due to insufficient data. We attempted to determine whether existing immunogenicity data could promote the identification of high-confidence neoantigens. DeepHLApan contains two independent parts (the binding model and the immunogenicity model), and its model framework consists of three layers of BiGRU with attention (Figure 1).

### The Prediction Performance of the Binding Model on Unseen Alleles

**Figure 2:**
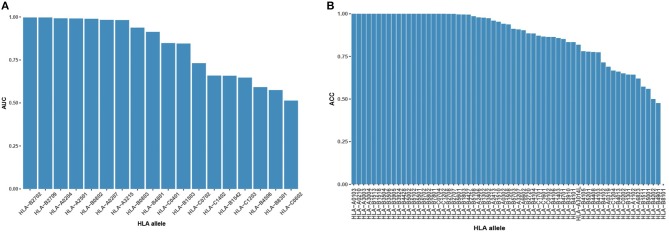
Binding model performance on never-seen HLA alleles. **(A)** Prediction AUC on alleles with both positive HLA-peptide pairs and negative pairs in descending order. **(B)** Prediction ACC on alleles with single-labeled HLA-peptide pairs in descending order.

During the binding model training, we created high-confidence pseudo HLA-peptide pairs to balance the training data of each allele and removed the HLA alleles with an imbalanced number of positive/negative pairs (see Materials and Methods). Of these removed alleles, 62 had pure positive HLA-peptide pairs, one had pure negative pairs, 14 had positive pairs more than 5-fold negative pairs and four had negative pairs of more than 5-fold positive pairs (Figure S2). We evaluated the performance of the binding model on these never-before-seen HLA alleles to discover its ability for pan-allele prediction. For the 18 alleles with both negative and positive pairs, most (9 out of 18, 50%) of them had AUC >0.9 (Figure 2A). For the rest of the untrained alleles, the majority (35 out of 63, 56%) had an accuracy >0.9 (Figure 2B). The reliable prediction results of the binding model on never-seen HLA alleles indicate that it is suitable for pan-allele prediction.

**Figure 3:**
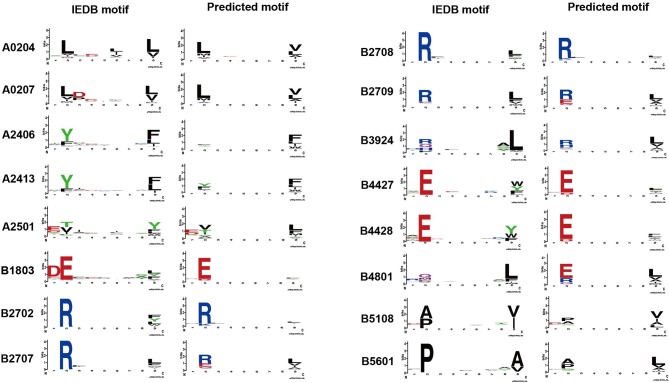
The comparison of actual motifs and predicted motifs on 16 HLA alleles. The motif logo is created by Weblogo. The actual motifs are based on their binding peptides, the predicted motifs are generated by taking top 1% predicted peptides out of 100,000 random peptides.

The binding model achieved good performance (AUC or accuracy >0.9) on 43 HLA alleles. To further investigate whether the binding model is able to learn truly correct motifs (only considering 9-mer) for these alleles, we compared the actual motifs and predicted motifs on 16 HLA alleles with more than 100 binding peptides (selected from 43 HLA alleles). The actual motifs were based on the binding peptides and the predicted motifs were generated by taking the top 1% predicted peptides out of 100,000 random peptides. WebLogo (49) was used for motif representation. We found that the predicted motifs are not exactly the same as the actual motifs, but they have similar patterns in most of the HLA alleles (Figure 3). The predicted motif of HLA-B48:01 is most dissimilar to the actual motif, but the amino acid arginine, glutamate, and glutamine are also conserved in the second amino acid residue of the predicted motif. These results demonstrate that the binding model of DeepHLApan does not simply boil down to a mix between many alleles of the training set, but has the ability to distinguish different alleles.

### The Binding Model Achieves Comparable Performance With Other HLA-Peptide Binding Prediction Tools

**Figure 4:**
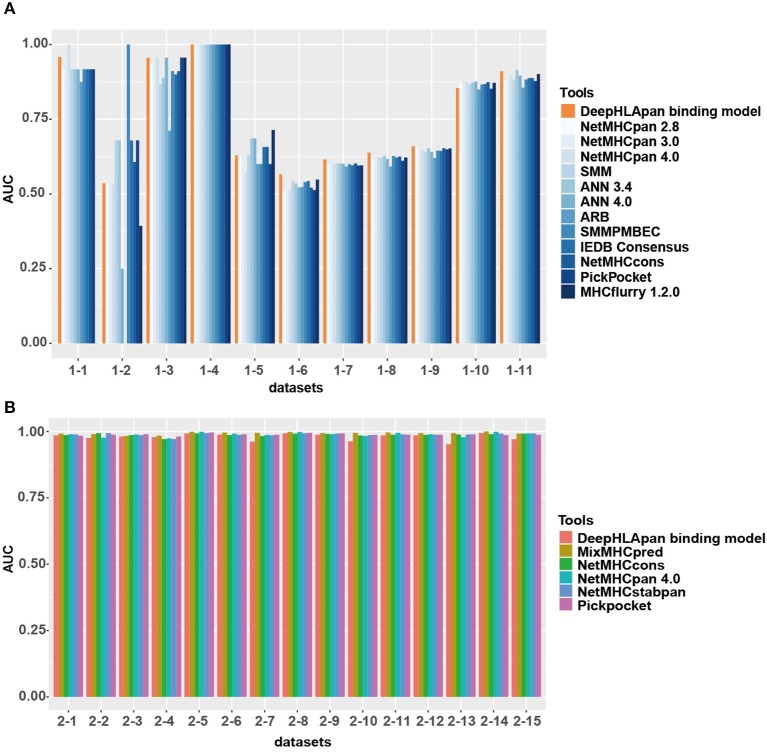
Model comparison between the binding model of DeepHLApan with other tools. **(A)** Performance of the binding model compared with the other 12 well-acknowledged tools on the latest IEDB benchmark datasets. **(B)** Performance of the binding model compared with the other 5 binding tools on the independent MS dataset. The detailed information of each sub-dataset is listed in Tables S7A,B.

The IEDB benchmark dataset is often used to compare the performance of different binding prediction tools. We used the latest IEDB benchmark dataset for this purpose. In the 11 sub-datasets that are suitable for all tools, the DeepHLApan binding model achieves the best performance for 6 out of the 11 (54.5%) sub-datasets while none of the other tools achieve a best performance in more than four sub-datasets (Figure 4A, Table S7A).

Interestingly, all the tools have a similar prediction ability in nearly all the sub-datasets. For instance, in sub-datasets 1, 3, 4, 10, and 11, all the tools achieved good performance, with some of them having an AUC of 1, which could be due to having enough corresponding HLA allele-specific HLA-peptide pairs in the training data. However, none of the tools could obtain excellent performance in sub-datasets 5, 6, 7, 8, and 9. Further investigation found that these datasets were derived from Liepe et al. (50), and one third of them contained binding information between spliced peptides and HLA alleles. Because all the tools were trained exclusively on natural (non-spliced) epitopes, the intrinsic differences between spliced and non-spliced antigenic peptides resulted in their limited prediction performance on spliced peptides (50).

We also collected an independent dataset from Mei et al. (39) for binding model evaluation (see Materials and Methods). Mei et al. provided a comprehensive analysis and benchmarking of 15 currently available tools for HLA-I peptide-binding prediction. The validation dataset they used has never been used for training in all previous developed tools and that's why only 5 tools [Pickpocket (51), MixMHCpred (25), NetMHCpan 4.0 (17), NetMHCcons (21), and NetMHCstabpan (52)] could obtain the AUC values in all datasets. After removing the sub-datasets that have been used for training in the binding model of DeepHLApan, 15 sub-datasets were retained and had been used for model comparison. The results showed that all tools achieved similar performance on these datasets while MixMHCpred performed slightly better than others (Figure 4B, Table S7B).

The methods focusing on HLA-peptide binding have been developed over many years. Currently available methods, such as MixMHCpred and NetMHCpan 4.0, have achieved excellent performance on the basis of available experimental data. Based on the performance comparison on the IEDB benchmarking dataset and the independent MS dataset, we concluded that the DeepHLApan binding model could obtain comparable performance with state-of-the-art HLA-peptide binding tools.

### The Immunogenicity Model Improves the Precision of Neoantigen Prediction Significantly

Because of the scarcity of immunogenicity data, few tools focus on the potential immunogenicity of pHLA, resulting in a high false positive rate (FPR) in practical applications. In this section, we explore whether existing immunogenicity data can contribute to neoantigen identification. Data used for immunogenicity model training were retrieved from the IEDB, and the validated neoantigens were retrieved from Koşaloǧlu-Yalçın et al. (40) (see Materials and Methods). In their study, NetMHCpan obtained remarkably good performance in terms of the AUC. However, the predicted precision on their datasets was 9.6 and 36.6% under the thresholds of 10 and 2%, respectively (Table 1). We added the predicted score from the immunogenicity model as an additional filter for neoantigen identification. The results showed that the predicted immunogenic score could improve the precision significantly (43.8 and 32.8% improvement under the thresholds of 10 and 2%, respectively) at the cost of less recall (Table 1, Table S8), which is acceptable for the purpose of predicting more reliable neoantigens rather than obtaining all potential neoantigens in practice. In addition, we also retrained an HLA-A02:01-restricted immunogenicity model to determine if more training data for each allele would attain both high precision and high recall due to the sufficient immunogenicity data of HLA-A02:01. The results showed that the HLA-A02:01-restricted model could improve the precision significantly (55.4 and 20.5% improvement under thresholds of 10 and 2%, respectively) and retain the recall with a decrease of <10% (Table 2, Table S9), indicating that the immunogenicity model could greatly contribute to high-confidence neoantigen identification with a growing amount of training data.

**Table 1:** The improvement of precision and decrease of recall with immunogenicity model on all available neoantigen predictions.

**Threshold (%)**	**NetMHCpan**		**NetMHCpan with immunogenicity model**	
	**Precision (%)**	**Recall (%)**	**Precision**	**Recall**
10	9.6	96	13.8% (+43.8%)	56.3% (−41.9%)
2	36.6	92.2	48.6% (+32.8%)	54.7% (−40.7%)

*The number in parentheses with a positive or negative sign indicates the percentage of improvement and decrease, respectively*.

**Table 2:** The improvement of precision and decrease of recall with immunogenicity model on HLA-A02:01-restricted neoantigen prediction.

**Threshold (%)**	**A02:01 restricted**		**A02:01 restricted with immunogenicity model**	
	**Precision (%)**	**Recall (%)**	**Precision**	**Recall**
10	10.1	96.0	15.7% (+55.4%)	88.0% (−8.3%)
2	37.5	84.0	45.2% (+20.5%)	76.0% (−9.5%)

*The number in parentheses with a positive or negative sign indicates the percentage of improvement and decrease, respectively*.

### Application of DeepHLApan on Published CD8^+^ T-Cell Epitopes

**Figure 5:**
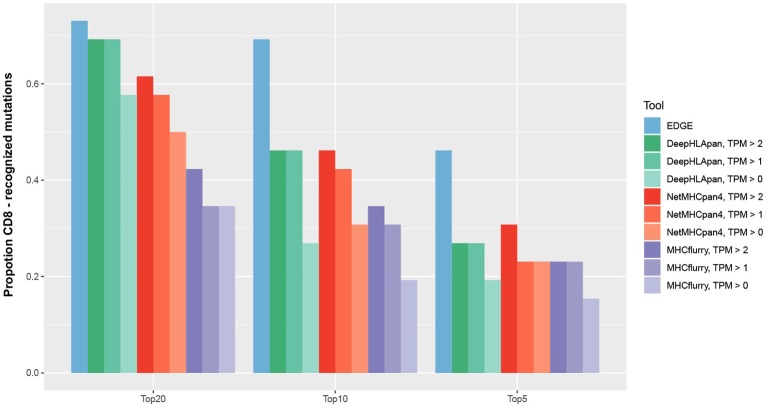
For 26 mutations with pre-existing T-cell responses, we ranked them in order of probability of presentation within their corresponding patients. The mutation rank of NetMHCpan 4.0 was measured by taking the minimum predicted rank across all mutation-spanning peptides. The number of predicted mutations ranked in the top 5, 10, and 20 by EDGE and MHCflurry were derived from Bulik-Sullivan et al. (27).

In recently published work (27), the EDGE model, which was trained by a tumor HLA-peptide MS dataset, achieved better performance on retrospective neoantigen T-cell data than MHCflurry (53), a tool for binding affinity prediction. To directly evaluate the performance of DeepHLApan on neoantigen prediction, we applied it on the same data they collected from four published works (41–44) (see Materials and Methods), which contained 2,023 mutations from 17 patients, 26 of which had pre-existing T-cell responses. We compared the performance of DeepHLApan with EDGE, MHCflurry and NetMHCpan 4.0 at the mutation level (Table S10). For each mutation, all 8–11 mer overlapping mutations were used for prediction and we ranked the mutations for each tool as described in the Materials and Methods. Taking the number of pre-existing T-cell responses in the 5, 10, or 20 top-ranked mutations for each patient as the measurement, DeepHLApan performed better than MHCflurry, but is comparable with NetMHCpan 4.0 for the 5 and 10 top-ranked mutations across different gene expression thresholds. With the 20 top-ranked mutations, DeepHLApan achieved better performance than NetMHCpan 4.0 and a comparable performance with EDGE at TPM >2 (Figure 5). Further evaluation of the AUC performance of four tools on each patient showed that EDGE performed best and DeepHLApan performed better than the other two tools on average under different thresholds of TPM (Table S11). We also evaluated the performance of DeepHLApan on the rank of the concrete HLA-peptide pairs within one patient. DeepHLApan ranked 32.2% (10/31) of the immunogenic HLA-peptide pairs as the 20 top-ranked HLA-peptide pairs at TPM >2 with 32.2% (10/31) missed due to predicted immunogenic scores <0.5 (Table S6). The rate could be 47.6% (10/21) if we ignored the missed HLA-peptide pairs and is better than that predicted by EDGE (27). All of the results mentioned above indicated that DeepHLApan could identify high-confidence neoantigens by filtering with an immunogenic score >0.5 and selecting HLA-peptide pairs with 20 top-ranked binding scores.

## Discussion

Neoantigens have been acknowledged as ideal targets for tumor immunogenicity and substantial effort has been made in neoantigen identification. However, most of the existing tools only consider the binding affinity between human leukocyte antigens (HLAs) and peptides and achieve unsatisfactory results. Recently, the EDGE model, which was trained by a tumor HLA peptide mass spectrometry dataset, achieved excellent performance in selecting high-confidence neoantigens (27). However, it did not consider the potential immunogenicity of predicted pHLA, as did many other tools, which cannot be ignored in the process of T-cell activation.

In this study, we propose a novel RNN-based method, DeepHLApan, for high-confidence neoantigen prediction considering both the possibility of mutant peptide presentation and the potential immunogenicity of pHLA. We demonstrate that the binding model could achieve good performance on unseen HLA alleles and has a comparable performance with other well-acknowledged tools on the latest IEDB benchmark datasets and an independent MS dataset. In the model comparison of the DeepHLApan binding model with other tools trained on datasets of canonical peptides, all of them performed poorly on the datasets with a high number of spliced peptides. One possible reason might be the different binding patterns between spliced and non-spliced antigenic peptides. Another reason might be that the identification of many of the spliced peptides stated in Liepe et al. (50) are ambiguous, and the vast majority of these peptides likely correspond to false-positives (54).

Using the immunogenicity model on the neoantigen datasets collected from Koşaloǧlu-Yalçın et al. we demonstrate that the predicted immunogenic score could significantly improve prediction precision of neoantigens. Although, with the improvement of precision of neoantigen identification by the immunogenicity model, the recall rate decreases in the neoantigen prediction, the cost is acceptable in clinical applications because the main problem in neoantigen identification is the high FPR for tumors with a lot of mutations. The decrease of recall can be solved as the amount of training data increases (Table 2). We also retain the predicted binding score for cases where some tumor types do not have enough mutations to tolerate poor recall. Finally, the application of DeepHLApan to the mutations with pre-existing T-cell responses shows that it has a performance comparable to that of the state-of-the-art EDGE model in high-confidence neoantigen prediction under the expression threshold of TPM >2.

There are also some limitations of our study. First, DeepHLApan does not have a significantly improved performance on the published CD8^+^ T-cell epitopes compared with EDGE. One of the possible reasons for this result is the limited number of immunogenic HLA-peptide pairs, which results in an immunogenicity model that is unable to classify all HLA-peptide pairs correctly. Another reason is the possibility that the datasets used for comparison are more suitable for EDGE. Another limitation of our study is that we consider all the HLA-peptide pairs as immunogenic if they have been validated to elicit T-cell activation at least once when training an immunogenic model. This assumption simplifies the evaluation of potential immunogenicity because whether the pHLA-matched TCR exists in the body is unknown. And with the alterations in the tumor microenvironment or immunoediting, the previous immunogenic neoantigens might be non-immunogenic. All these complex factors should be considered if we want to obtain accurate prediction. One of the solutions to this issue, and a direction for future research, would be to develop a model based on the pHLA-TCR pairs for more reliable immunogenicity prediction for specific individual. With that model, we could predict higher confidence neoantigens by taking advantage of whole-exon sequencing (call mutations) and RNA-Seq sequencing [evaluate gene expression and analyze the complementarity-determining regions 3 [CDR3] sequences by TRUST (55)] or TCR sequencing (call CDR3 sequences).

## Data Availability Statement

Publicly available datasets were analyzed in this study. This data can be found here: http://www.iedb.org/, http://www.ensembl.org.

## Author Contributions

ZZ and SC conceived and supervised the study. JiaW and ZZ designed the tool. JinW, WW, and JZ wrote the scripts and trained the models. JinW, BZ, and WZ performed the data analysis. ZS, XG, and JiaW participated in designing and developing the algorithms. ZZ, JinW, and BZ drafted the manuscript. All authors have read and approved the final manuscript.

### Conflict of Interest

The authors declare that the research was conducted in the absence of any commercial or financial relationships that could be construed as a potential conflict of interest. The reviewer SG and handling editor declared their shared affiliation.

## Supplementary Material

Figure S1 The distribution of binding data collected from the IEDB before and after data balance. **(a)** Before data balance, the distribution of 327,178 non-redundant HLA-peptide pairs, which covers 169 HLA alleles. The HLA alleles are displayed clockwise according to the HLA-peptide pairs they possess. The length of each HLA allele is proportionable to the number of HLA-peptide pairs. Red and green indicates positive and negative pairs, respectively. The histogram illustrates the length distribution of peptides (ranges from 8 to 15) of each HLA allele. **(b)** After data balance, the distribution of 437,077 HLA-pairs covering 81 HLA alleles. Only alleles with more than 3,000 HLA-peptide pairs are labeled.

Figure S2 The HLA-peptide pair distribution of the removed alleles. These removed alleles are divided into four types: 62 had pure positive HLA-peptide pairs, one had pure negative pairs, 14 had positive pairs more than 5-fold of the negative pairs and four had negative pairs more than 5-fold of the positive pairs. The number in each cell represents the number of HLA-peptide pairs of each HLA allele.

Table S1 The binding data collected from the IEDB. HLA-peptide without affinity are set to 0 in the “Affinity” column.

Table S2 The detailed information of the removed alleles with their predicted binding scores and labels. In the “label” column, 0 stands for non-binding, and 1 stands for binding.

Table S3 The binding data used for training the final binding model.

Table S4 The immunogenicity data collected from the IEDB.

Table S5 **(A)** HLA-peptide pairs created by the data from Tran et al., Gros et al., and Zacharakis et al. with their predicted binding scores and immunogenic scores. **(B)** HLA-peptide pairs created by the data from T Stronen et al. with their predicted binding scores and immunogenic scores.

Table S6 Immunogenic HLA-peptide pairs derived from Gros et al., Tran et al., and Stronen et al. with their ranks predicted by DeepHLApan under TPM>2.

Table S7 **(A)** The AUC performance of the binding model of DeepHLApan on the latest IEDB benchmark datasets compared with other tools. The best performance of each sub-dataset is highlighted in red. **(B)** The AUC performance of the binding model of DeepHLApan on the datasets collected from Mei et al. compared with other tools. The best performance of each sub-dataset is highlighted in red.

Table S8 The validated neoantigens retrieved from Koşaloǧlu-Yalçın et al. and the corresponding predicted immunogenic scores.

Table S9 HLA-A02:01-restricted validated neoantigens retrieved from Koşaloǧlu-Yalçın et al. and the immunogenic scores predicted by the HLA-A02:01-restricted model.

Table S10 The predicted mutation score of four tools at TPM>0. Smaller the values of MHCflurry and NeMHCpan4 are and larger the values of EDGE and DeepHLAPan are, stronger the binding probabilities.

Table S11 The performance (AUC) of 4 tools on neoantingen prediction for each patient at different cut off of TPM. The best performance of each patient is highlighted in bold.
