# Supplementary figures and images for: DeepHLApan: A Deep Learning Approach for Neoantigen Prediction Considering Both HLA-Peptide Binding and Immunogenicity
Source: Front Immunol. 2019 Nov 1;10:2559. doi: 10.3389/fimmu.2019.02559 (PMC6838785; doi:10.3389/fimmu.2019.02559)

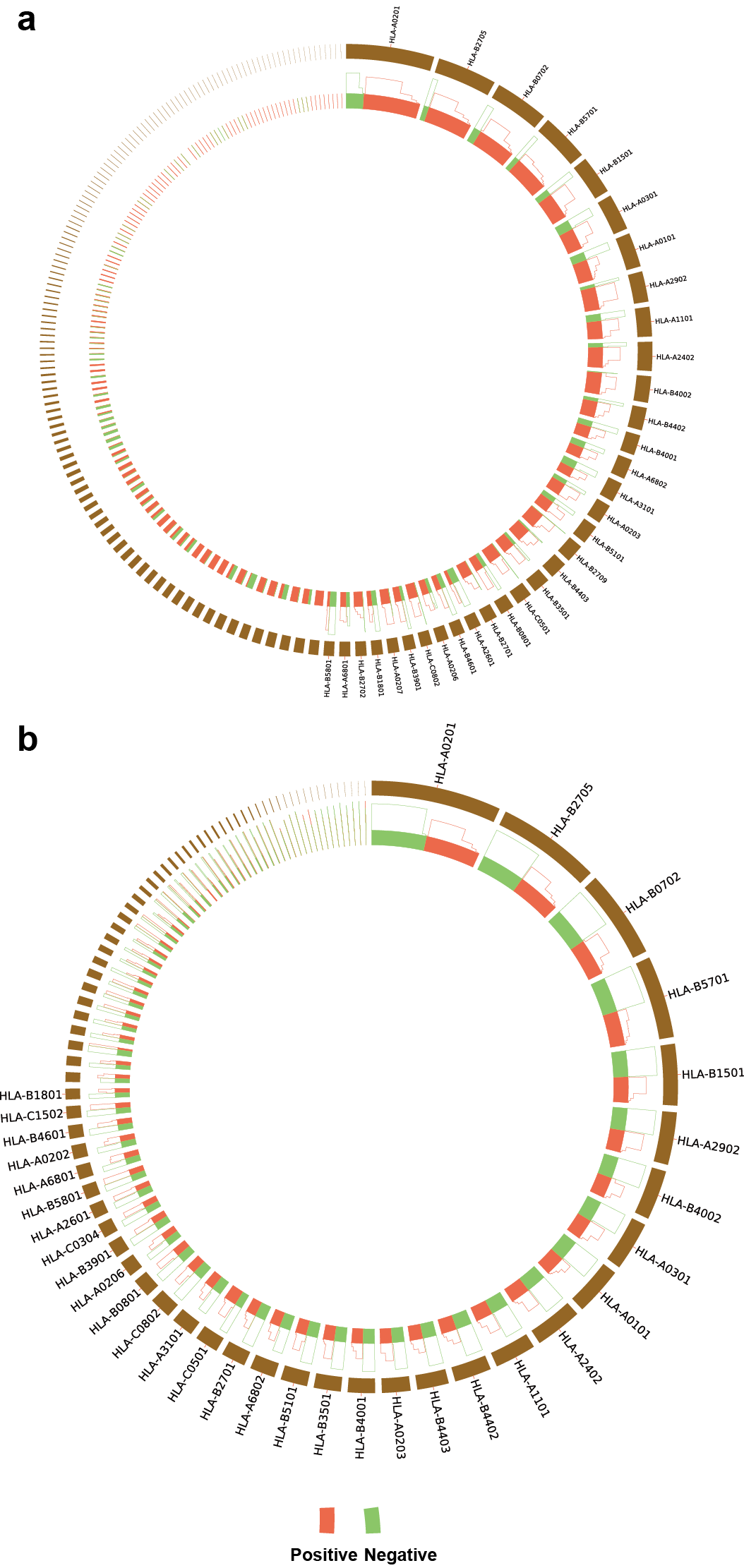

Supplement: Figure S1 — The distribution of binding data collected from the IEDB before and after data balance. (a) Before data balance, the distribution of 327,178 non-redundant HLA-peptide pairs, which covers 169 HLA alleles. The HLA alleles are displayed clockwise according to the HLA-peptide pairs they possess. The length of each HLA allele is proportionable to the number of HLA-peptide pairs. Red and green indicates positive and negative pairs, respectively. The histogram illustrates the length distribution of peptides (ranges from 8 to 15) of each HLA allele. (b) After data balance, the distribution of 437,077 HLA-pairs covering 81 HLA alleles. Only alleles with more than 3,000 HLA-peptide pairs are labeled. [file Image_1.JPEG]

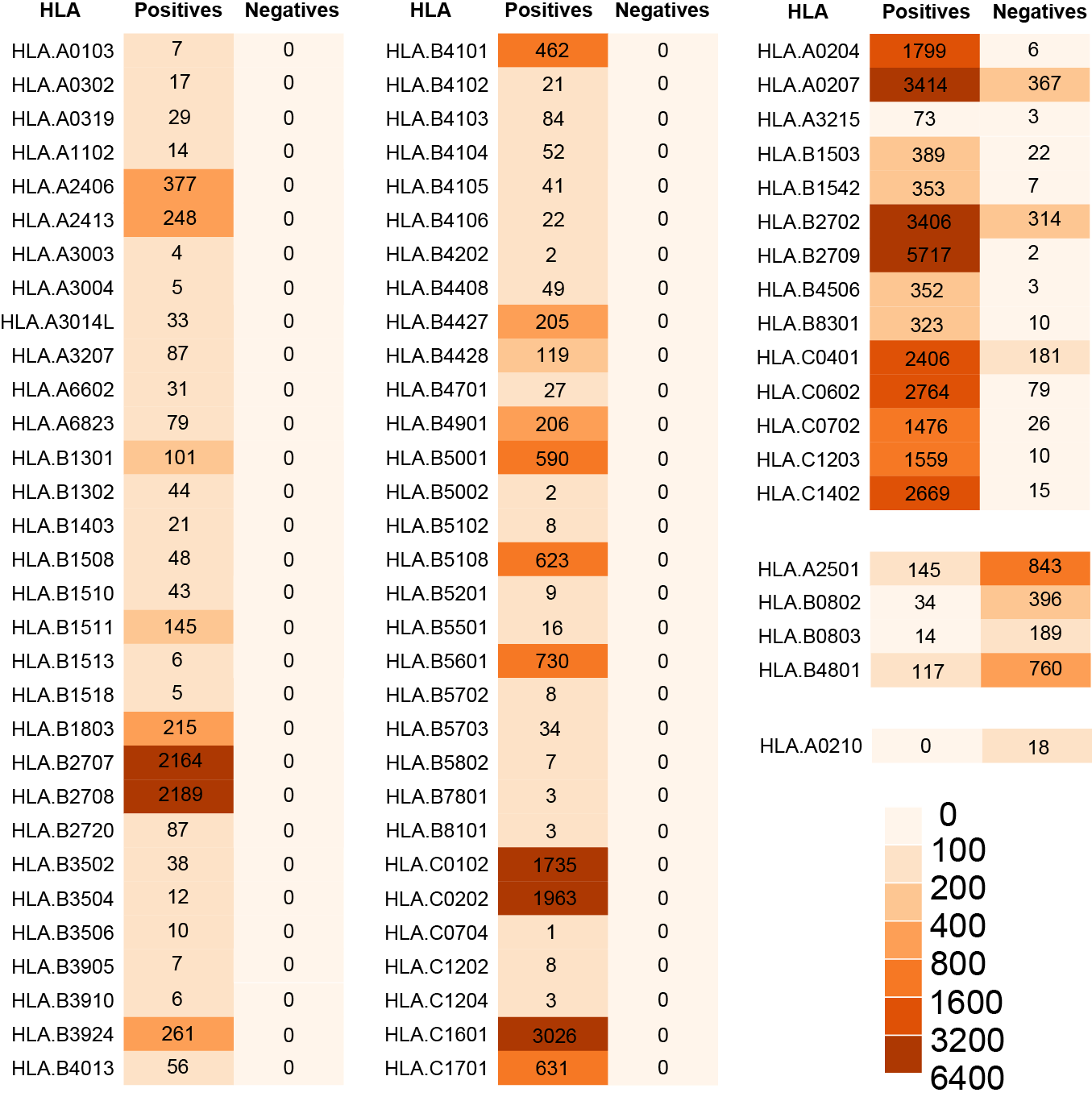

Supplement: Figure S2 — The HLA-peptide pair distribution of the removed alleles. These removed alleles are divided into four types: 62 had pure positive HLA-peptide pairs, one had pure negative pairs, 14 had positive pairs more than 5-fold of the negative pairs and four had negative pairs more than 5-fold of the positive pairs. The number in each cell represents the number of HLA-peptide pairs of each HLA allele. [file Image_2.JPEG]
